# Screening and Identification of Antipyretic Components in the Postfrost Leaves of *Morus alba* Based on Multivariable and Continuous-Index Spectrum-Effect Correlation

**DOI:** 10.1155/2019/8796276

**Published:** 2019-10-16

**Authors:** Yongsheng Qu, Liang Wang, Wei Guo

**Affiliations:** Shandong Academy of Chinese Medicine, 7 Yanzishanxi Street, Jinan 250014, China

## Abstract

The leaves of *Morus alba* (LMA) are crucial traditional Chinese medicine (TCM) of clearing heat. In ancient Chinese materia medica and the current Pharmacopoeia of the People's Republic of China, LMA are recorded to be harvested after frost for medicinal purpose. However, the reason and mechanism of this traditional usage have been still unknown so far. In this work, it was confirmed firstly that the antipyretic effect of LMA after frost was better than that of before frost significantly on feverish rats. Subsequently, the chemical profiles of LMA before and after frost were characterized by fingerprint, respectively. Then, the endemic peaks after frost and positive differential peaks were screened as the research object of spectrum-effect correlation by orthogonal signal correction partial least square discrimination (OPLS). Finally, a multivariable and continuous-index spectrum-effect correlation model coupled with OPLS was established. As a result, the antipyretic components of postfrost LMA were screened and identified as citric acid derivative and tryptophan which may be the synergistic material basis. The study can provide a scientific foundation for the enhancement of effects in the postfrost LMA. Moreover, the strategy of this research could provide a valuable reference for revealing the material basis of synergetic or antagonistic effects among other complex drug systems.

## 1. Introduction

*Morus alba* L. is an essential economic crop worldwide and a rapid-growing woody plant. LMA can be used for making tea and noodles. And it is also a kind of popular TCM, often used for dispelling wind, clearing heat, clearing lung, and moisturizing dryness [1]. It contains abundant secondary metabolites, such as polyphenols, flavonoids, and alkaloids [2], which exhibit many bioactivities, such as hypoglycemic, antioxidant, anti-inflammatory, and antipyretic activity [3]. Qin and Ma found that LMA showed certain synergistic actions on relieving fever [4]. However, its antipyretic components remain unclear and still need to be further studied. Until today, there have only been a small number of reports on the antipyretic material of LMA; hence, it is necessary to further study the antipyretic material bases to provide scientific evidence for quality control and safe usage.

LMA is officially recorded to be harvested after frost in Pharmacopoeia of the People's Republic of China (2015 edition) [1]. Frost is commonly regarded as a solar term which is named Frost's Descent in the traditional Chinese calendar. The quality of postfrost LMA has been considered more excellent than prefrost LMA in clinic from ancient to the present. In this work, the differences of antipyretic effect between postfrost and prefrost LMA were evaluated in yeast-induced feverish rats. The continuous index of temperature curve was recorded. As a result, the antipyretic effect of postfrost LMA was better than that of prefrost LMA significantly. Accordingly, it is speculated that frost may lead to the change of chemical components, and the positive differential components may be the key reason to result in the enhancement of antipyretic effect. Thus, the change of chemical profile based on the traditional antipyretic effect was regarded as the research object in the further study.

Due to the complicated constituents in TCM, it is a challenging task to find the changed components that are responsible for the differences between samples. Fingerprint is a widely used technology to characterize the chemical profile in traditional Chinese medicine (TCM). And OPLS, which can evaluate the change degree of the components, was used to extract the positive changed components as variables for the subsequent spectrum-effect correlation study. Thus, fingerprint in conjunction with OPLS was explored to screen the targets from complex matrix samples.

The spectrum-effect correlation technique, as one of the key techniques of modern TCM research, is a good approach to predict the possible active compounds [5]. The statistical analysis method of spectrum-effect correlation research includes multiple linear regression, bivariate correlation analysis, grey relational analysis, partial least squares (PLS), and artificial neural network. These methods apply to the different types of data. How to choose appropriate method depends on the feature of data. Generally, OPLS and PLS are suitable for the analysis of multidependent variables and multi-independent variables. OPLS is an improved PLS being capable of eliminating the uncorrelated data and improving the explanatory ability and accuracy of the model [6], which can make the best use of obtained data and predict accurately. Therefore, it is an effective approach to study the spectrum-effect correlation owing to the above virtues [7].

In this study, in order to present dynamic changed process accurately and efficiently, the negative changed common peaks were excluded firstly in OPLS analysis. Only the endemic peaks of postfrost, positive changed common peaks were regarded as the research object of spectrum-effect correlation. Then, the multivariable and continuous-index spectrum-effect correlation coupled with OPLS was developed to elucidate and screen potential antipyretic components, which may also lead to references for similar work in synergetic or antagonistic effects of other TCMs.

## 2. Materials and Methods

### 2.1. Samples, Reagents, and Animals

Ten samples were harvested, respectively, before and after frost in Xiajin County, Shandong Province, China, which were authenticated as the leaves of *Morus alba* by Professor Huibin Lin (Shandong Academy of Chinese Medicine, Jinan, China). The same batch of prefrost and postfrost samples was collected from the same plant (Table 1). Voucher specimens were preserved in the herbarium of Shandong Academy of Chinese Medicine. Methanol, acetonitrile, and phosphoric acid used for HPLC analysis were of chromatographic grade (Merck, Darmstadt, Germany). Deionized water (18.2 MΩ) was prepared using a Milli-Q system (Millipore, Billerica, USA). Other reagents were of analytical grade. Baker's yeast (*Saccharomyces cerevisiae*) was purchased from Angel Yeast Co., Ltd. (Yichang, China, No. CE20180130WE35). Saline was purchased from Shandong Qidu Pharmaceutical Co., Ltd. (Zibo, China, No. H37020764). Acetaminophen tablets were purchased from Northeast Pharm (Shengyang, China, No. H21020448).

Male Wistar rats weighing 150–170 g were supplied by Pengyue Laboratory Animal Co., Ltd. (approval No. SCXK (Lu) 2014-0007. Jinan, Shandong Province, China). The Animal Ethics Committee of Shandong Academy of Chinese Medicine approved all animal protocols. The animal experiments were carried out according to the Guide for the Care and Use of Laboratory Animals.

### 2.2. Apparatus and Conditions

The Agilent 1200 series (Agilent Technologies, Palo Alto, CA, USA) consisting of a G1322A degasser, a G1311A quaternary pump, a G1316A thermostatted column compartment, a G1329A automatic sampler, a G1330B ALS Therm, a G1315B diode array detector, and the ChemStation software (version B01.03) was employed for the sample analysis.

HPLC fingerprints were performed on an Agilent SB-C18 reverse-phase column (250 mm × 4.6 mm, 5.0 *μ*m) coupled with a guard column (Phenomenex, Torrance, CA, USA). Mobile phases consisted of water containing 0.3% phosphoric acid (A), methanol (B), and acetonitrile (C). The gradient elution: 0–120 min, 1% B; 0–10 min, 0% C; 10–40 min, 0–6% C; 40–60 min, 6–8% C; 60–90 min, 8–15% C; and 90–120 min, 15% C. Column temperature, 30°C; flow rate, 1.0 mL/min; and detection wavelength, 240 nm.

The ESI-MS^*n*^ detection was performed with an Agilent 6320 Ion Trap LC/MS. The MSD was controlled, and total ion chromatograms and mass spectra were recorded using the LC/MSD Trap software 5.3. The experiment was performed on negative electrospray ionization mode (−ESI) and positive electrospray ionization mode (+ESI). Optimized MS parameters were set as follows: mass range, 50 to 1000 Da; autoMSn depth, 5; scan speed, 26 000 m/z/s; nebulizer pressure, 35 psi; dry gas temperature, 350°C; dry gas flow rate, 9 L/min; capillary voltage, 4 000 V; split ratio, 4 : 1.

The ESI-TOF-MS detection was performed on a Xevo G2-S Q-TOF MS system (Waters Corporation, MA, USA). The experiment was detected on both ESI (‒) ionization modes. Optimized ESI source parameters were set as follows: mass range, 50 to 1500 Da; scan time, 0.2 s; high collision energy, 30 to 50 V; capillary voltage, 500 V; sampling cone voltage, 40 V; source temperature, 100°C; dry gas temperature, 450°C; dry gas flow rate, 900 L/h; and cone gas flow, 50 L/h. Data acquisition was controlled by MassLynx V4.1 software (Waters Corporation, MA, USA).

### 2.3. Preparation of Samples Solution

Samples solution for the HPLC fingerprinting analysis (S1): the powdered leaves of *Morus alba* (1 g) were extracted by refluxing with heating water (50 mL) for 1 h, cooled to room temperature, and made up for lost weight. The extract was filtered through a 0.45 *μ*m filter membrane for the HPLC fingerprinting analysis.

Samples solution for administration (S2): the powdered leaves of *Morus alba* (5 g) were extracted by refluxing with heating water (50 mL, twice) for 1 h and filtered. Then, the filtrates were combined and concentrated to 50 mL under vacuum. The concentrated solution was used for administration.

Acetaminophen solution (S3): 0.3 g of acetaminophen tablets was dissolved by using 55.6 mL water to obtain 0.0054 g/mL solution.

20% yeast solution (S4): 56 g of yeast was grounded into fine powder and dissolved by using 280 mL water, mixed well before use.

### 2.4. HPLC Fingerprint Analysis Method Validation

The HPLC fingerprint analysis method was validated using parameters such as precision, reproducibility, and stability of sample. The precision was determined by analyzing one sample six times continuously. The reproducibility was carried out using six independent sample solutions. The stability of sample was determined by analyzing one sample at 0 h, 1 h, 2 h, 4 h, 12 h, and 24 h, respectively.

### 2.5. Animal Experiments

All rats were acclimated for 7 days in a controlled room with temperature (23 ± 2°C), humidity (60 ± 5%), and light/dark conditions altered for each 12 h every day. The rats received a standard diet and water *ad libitum*. During the following 3 days, the rectal temperatures were measured twice per day using a digital thermometer for the regular rhythm of body temperature, and rats whose body temperature fluctuations were under 0.3°C were selected for the formal study.

One-hundred and thirty-eight rats were randomly divided into 23 groups (6 rats for each), including a control group (no injection, 10 mL/kg saline i.g.), model group (subcutaneous injection of S4, 10 mL/kg saline i.g.), an acetaminophen group (subcutaneous injection of S4, 10 mL/kg S3 i.g.), 10 prefrost administrated groups, and 10 postfrost administrated groups (subcutaneous injection of S4, 10 mL/kg corresponding S2 i.g.). Firstly, the rectal temperatures were measured 5 times (an hour for each interval) as basal body temperature, and weights were also measured. Then, the rats of all groups except for the control group were subcutaneously injected with 10 mL/kg S4 in their back. The same dose of saline was given to the control group instead. After 6.5 h of the S4 injection, a dose of 10 mL/kg S2 was given to the corresponding administrated groups, and a dose of 10 mL/kg S3 was given to the acetaminophen group. The administrative doses were calculated according to clinical dose on humans. Simultaneously, the rectal temperatures were begun to measure with the interval of an hour, 14 times altogether.

### 2.6. Data Handling

“Chinese traditional medicine chromatographic fingerprint similarity evaluation system (2004, 1.0 A Edition)” was used to analyze global similarity of prefrost and postfrost fingerprint. Multipoint correction of chromatographic peak position was performed to align peaks. A standard chromatogram was generated by the average method. The common peaks and the endemic peaks of prefrost and postfrost were recognized. The common peaks were performed by OPLS using Par scaling to screen differential peaks, and then the negative common peaks were excluded. The endemic peaks of postfrost and positive differential common peaks made up the fingerprint data matrix. Then, the matrix and the antipyretic data (8.5–15.5 h) were imported into the SIMCA (Umetrics, Umea, Sweden) software to study the spectrum-toxicity relationship.

Statistical evaluations of the peak areas of the key differential components between prefrost and postfrost samples were analyzed by independent *t*-test. The probability level of *p* < 0.05 was considered to be significant in the analyses.

## 3. Results

### 3.1. Antipyretic Effects

**Figure 1:**
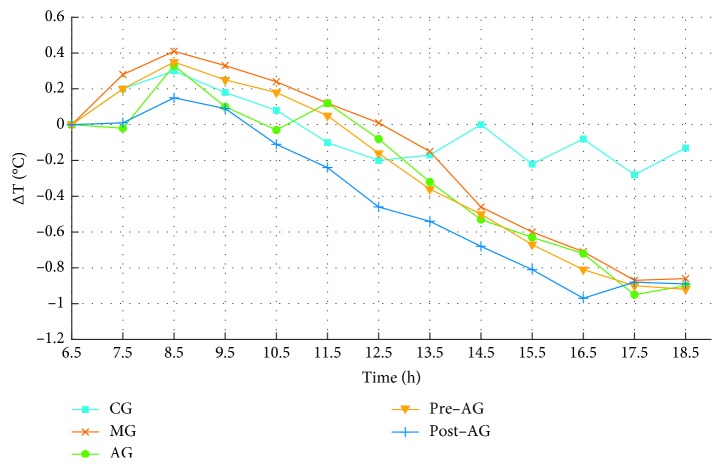
The temperature curve of prefrost and postfrost samples.

The rectal temperatures of rats in each group before and after drug administration were recorded to monitor the body temperature changes. Rectal temperatures were measured at 6.5, 7.5, 8.5, 9.5, 10.5, 11.5, 12.5, 13.5, 14.5, 15.5, 16.5, 17.5, and 18.5 h after the injection of 20% yeast solution. Taking time as independent variables, Δ*T* (*T*−*T*_6,5 h_) as dependent variables, draw a temperature curve, which is shown in Figure 1. The temperature fluctuations of control group (CG) changed slightly. The rectal temperatures of model group (MG) were higher than the temperatures of other groups from 8.5 h to 18.5 h (except for 13.5 h), indicating that the yeast-induced feverish model was successful. After drug administration at 6.5 h, the rectal temperatures of MG, acetaminophen group (AG), prefrost administrated groups (pre-AG), and postfrost administrated groups (post-AG) increased firstly, reached the highest at the time-point of 8.5 h, and then decreased (Table 2). The body temperatures from 7.5 h to 16.5 h were significantly different between post-AG and MG (*p* < 0.01), while there were no significant differences between pre-AG and MG. It was indicated that post-AG had a better antipyretic effect than that of pre-AG. In addition, after 8.5 h, AG continued to drop for 2 h and then began to rise, while the temperature of post-AG kept a downward trend for 10 h and finally maintained similar antipyretic effect compared with AG at 17.5 h, which indicated that the antipyretic effect of post-AG was more sustained and stable than that of AG.

### 3.2. Fingerprint Analysis

Different mobile phases including acetonitrile-water, methanol-water, acetonitrile-water with 0.3% phosphoric acid, and methanol-water with 0.3% phosphoric acid were tried. The results showed that binary solvent systems could not obtain the satisfied results. Thus, the ternary systems of 0.3% phosphoric acid-acetonitrile-methanol was tested and chosen as the most appropriate mobile phase of fingerprint analysis. The method validation results of fingerprint established showed that the relative standard deviations of relative retention times for major chromatographic peaks were less than 1%, and the relative standard deviations of relative peak areas were less than 3%. It was indicated that the precision of instrument and the reproducibility of extraction method was good, and the sample was stable in 24 h.

**Figure 2:**
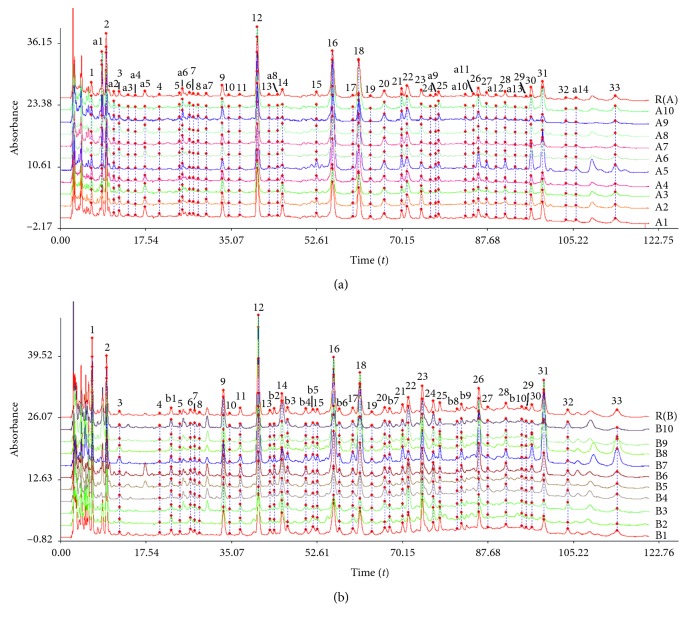
The fingerprints of prefrost and postfrost samples. (a) Prefrost. (b) Postfrost.

**Figure 3:**
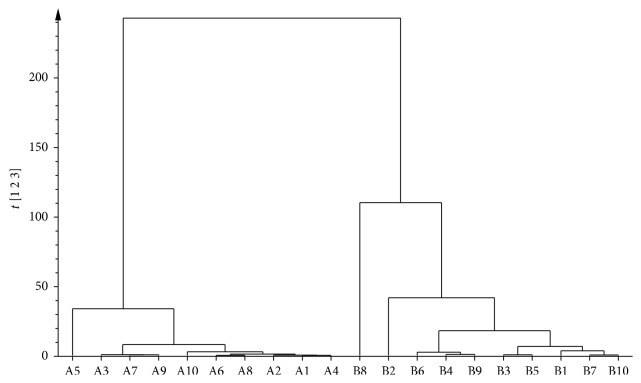
The hierarchical clustering plot of prefrost and postfrost samples.

The common peaks of prefrost and postfrost samples were 33 (peak no.: 1–33). The endemic peaks of prefrost samples were 14 (peak no.: a1–a14), and the endemic peaks of postfrost samples were 10 (peak no.: b1–b10), as shown in Figure 2. The similarities of the twenty samples to the standard fingerprint were 0.759–0.972, while the similarities of the ten prefrost samples to the standard fingerprint were 0.936–0.994 and the similarities of the ten postfrost samples to the standard fingerprint were 0.863–0.995. It was indicated that the changed chemical profiles after frost were obvious. And the results of hierarchical clustering showed that prefrost and postfrost samples were divided into two groups, respectively (Figure 3). It was further verified that prefrost and postfrost samples have significant difference.

### 3.3. Identification of Compounds from LMA Fingerprint

The identification of common peaks was carried out by comparing and combining analysis data of ESI-MS^*n*^ and ESI-TOF-MS. Fragmentation characteristics of common peaks was obtained by HPLC-ESI-MS^*n*^ detection in negative and positive modes, respectively. The exact masses were obtained, and the molecular formulas of compounds were calculated by HPLC-ESI-TOF-MS. The obtained MS data and identification results of common peaks are summarized in Table 3.

### 3.4. Differential Analysis

**Figure 4:**
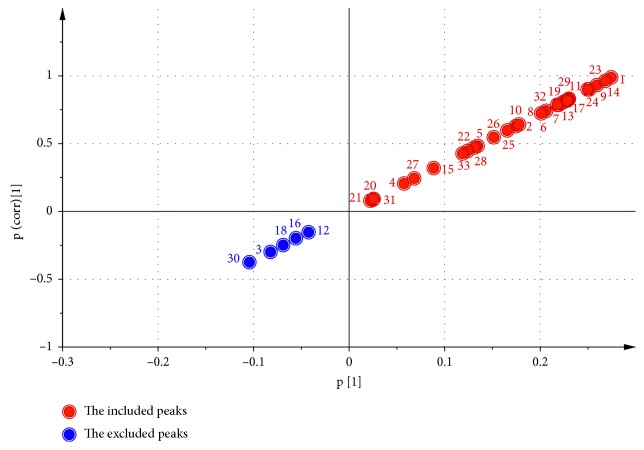
The S-plot of peaks.

The common peaks were performed by OPLS to screen differential peaks. The negative changed common peaks (Peak 12, 16, 18, 3, 30) in the third quadrant were excluded. The peaks in the first quadrant were the positive changed common peaks for the further spectrum-effect correlation analysis (Figure 4).

### 3.5. Spectrum-Effect Correlation Analysis

Generally, spectrum-effect correlation analysis between multivariable dependent variables and multivariable independent variables is performed by OPLS or PLS. OPLS is an improved PLS that removes irrelevant information to dependent variables in predicted matrix, which can offer enhanced model interpretation and is more favorable to finding out the correlated and uncorrelated variables during the targeted process. It can provide the correlation of chromatogram peaks and its effect accurately and estimate how much *X*-variables contributed to the correlation with *Y*-variables, thereby singling out the corresponding active components. OPLS provided the results with scatter plots and score plots, which visualized the analytical results. This OPLS-based method was more accurate and visual-friendly for screening the antipyretic components. The previous literatures usually analyze the correlation of main common peaks with effect, which cannot present the decrease and increase of the effect. In this study, we excluded the negative differential common peaks and took the endemic peaks of postfrost and positive differential common peaks as the research object of spectrum-effect correlation creatively, which can reveal the synergism after frost of LMA roundly and accurately.

**Figure 5:**
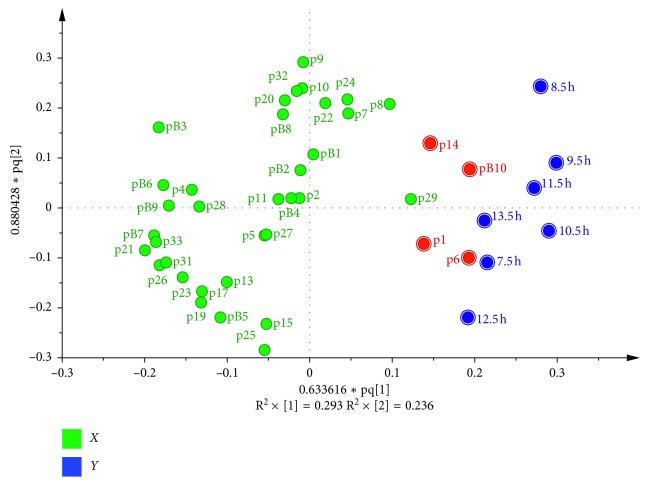
The loading scatter plot.

The loading scatter plot (Figure 5) displayed the relation between *X*-variables and *Y*-variables. In the present work, it was used to screen compounds (*X*-variables) correlated to antipyretic effect (*Y*-variable). The *Y*-variable was on the right of the *y* axis. Thus, the *X*-variables near *Y*-variable were correlated with the *Y*-variable in a positive manner. In addition, the further an *X*-variable is from the origin of the coordinate, the better it is connected to the *Y*-variable. In view of this, several *X*-variables (b10, 1, 6, and 14) which were shown in red were preliminarily chosen for further screening.

**Figure 6:**
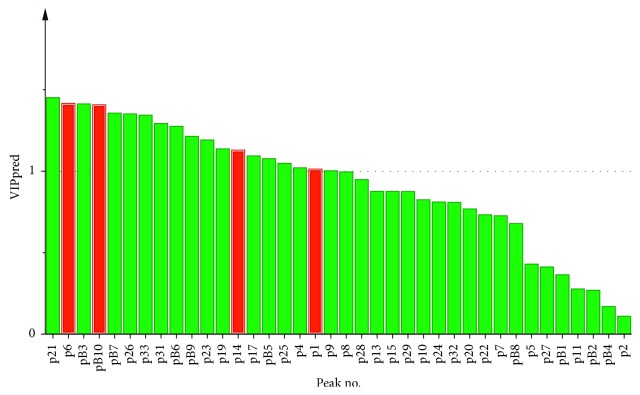
The plot of VIP.

**Figure 7:**
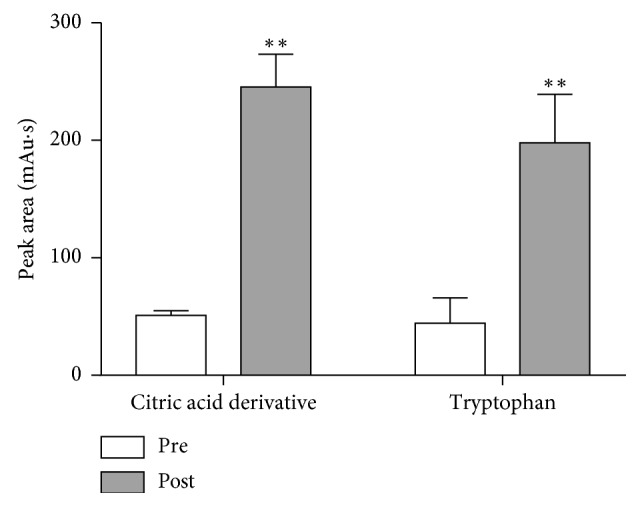
The hierarchical clustering plot of prefrost and postfrost samples.

The plot of variable importance in projection (VIP) expresses VIP value in the gradually decreased order. The VIP value is commonly applied to estimate how much *X*-variables contributed to the correlation with *Y*-variables. The variable with VIP value larger than 1 indicates it is statistically significance. Then, taking the results of loading scatter plot findings into account, the variables (b10, 1, 6, and 14) were determined to be positively correlated to antipyretic effect. These peaks were coloured in red (Figure 6). As seen from Figure 2, the areas of peak B10 and peak 6 were too small to identify. Peak 1 and peak 14's areas were large, their VIP values were larger than 1, and in the loading scatter plot, they were positively correlated to antipyretic effect. Based on the above reasons, the key components increasing the antipyretic effect in LMA after frost were the components of peak 1 and peak 14, which was speculated as citric acid derivative [11] and tryptophan [12] in Table 3, respectively. The independent *t*-test results showed that the two compounds before and after frost were statistically significant (Figure 7).

Tryptophan, as an important amino acid, has the function of nourishing nerve and reducing excitotoxicity. When the rats were injected with yeast, the levels of quinolic acid upregulated [18], and quinolic acid produced tryptophan degradation through the kynurenine pathway [19]. The tryptophan content of LMA was increased significantly after frost, which could supplement the degradation of tryptophan and correct the disorder state induced by fever in rats.

As shown in Figure 4, neochlorogenic acid, chlorogenic acid, 4-caffeolyquinic acid, and rutin decreased after frost in LMA, while citric acid derivative and tryptophan increased significantly after frost. Simultaneously, according to the result of spectrum-effect study, citric acid derivative and tryptophan could also be the key components for the enhancement of effects in the postfrost LMA. The consistent result of these two parts could support each other, which may illustrate the credibility of this study.

## 4. Conclusions

In this work, a spectrum-antipyretic effect correlation in LMA was first studied. Distinguished from the previous study of spectrum-effect correlation, the key feature of the present study was the establishment of multivariable and continuous-index spectrum-effect correlation base on the fingerprint change of prefrost and postfrost and the enhancement of antipyretic effects. Taking the endemic peaks of postfrost and positive differential common peaks as the research object of spectrum-effect correlation creatively, it was mainly focused on dynamic change of chemical profile and curative effects. The results showed that the chemical profile of samples before and after frost have significant difference. Postfrost had better antipyretic efficacy than prefrost. The synergistic material basis of frosting that contributed to the difference of antipyretic effects in postfrost was also found. In a word, our study can provide a scientific foundation for the enhancement of effects in LMA after frost. Moreover, the strategy of this research could provide a valuable reference for revealing the material basis of synergetic or antagonistic effects among other TCMs or complex drug systems.

## Figures and Tables

**Table 1:** Information of samples.

	No. of samples	No. of plants
Prefrost	A1	1
	A2	2
	A3	3
	A4	4
	A5	5
	A6	6
	A7	7
	A8	8
	A9	9
	A10	10

Postfrost	B1	1
	B2	2
	B3	3
	B4	4
	B5	5
	B6	6
	B7	7
	B8	8
	B9	9
	B10	10

**Table 2:** The antipyretic data of prefrost and postfrost samples ($\bar{X}\pm s$, *n* = 6).

Time (h)	CG	MG	AG	Pre-AG	Post-AG
6.5	0.00	0.00	0.00	0.00	0.00
7.5	0.20 ± 0.21	0.28 ± 0.20	−0.02 ± 0.33	0.20 ± 0.15	0.01 ± 0.27^*∗∗*^
8.5	0.30 ± 0.20	0.41 ± 0.29	0.33 ± 0.27	0.35 ± 0.11	0.15 ± 0.18^*∗∗*^
9.5	0.18 ± 0.49	0.33 ± 0.37	0.10 ± 0.33^*∗∗*^	0.25 ± 0.07	0.09 ± 0.19^*∗∗*^
10.5	0.08 ± 0.37	0.24 ± 0.40	−0.03 ± 0.36^*∗*^	0.18 ± 0.13	−0.11 ± 0.21^*∗∗*^
11.5	−0.10 ± 0.29	0.12 ± 0.34	0.12 ± 0.41	0.05 ± 0.20	−0.24 ± 0.23^*∗∗*^
12.5	−0.20 ± 0.50	0.01 ± 0.59	−0.08 ± 0.75	−0.16 ± 0.16	−0.46 ± 0.26^*∗∗*^
13.5	−0.17 ± 0.29	−0.15 ± 0.34	−0.32 ± 0.4	−0.36 ± 0.16	−0.54 ± 0.24^*∗∗*^
14.5	0.00 ± 0.35	−0.46 ± 0.38	−0.53 ± 0.36^*∗*^	−0.50 ± 0.20	−0.68 ± 0.18^*∗∗*^
15.5	−0.22 ± 0.31	−0.60 ± 0.22	−0.63 ± 0.35	−0.67 ± 0.17	−0.81 ± 0.14^*∗∗*^
16.5	−0.08 ± 0.34	−0.71 ± 0.17	−0.72 ± 0.28	−0.81 ± 0.13	−0.97 ± 0.22^*∗∗*^
17.5	−0.28 ± 0.32	−0.87 ± 0.25	−0.95 ± 0.34	−0.90 ± 0.24	−0.88 ± 0.19
18.5	−0.13 ± 0.31	−0.86 ± 0.23	−0.9 ± 0.33	−0.92 ± 0.21	−0.89 ± 0.10

^*∗*^ *p* < 0.05 and ^*∗∗*^*p* < 0.01, compared to MG, respectively.

**Table 3:** Compounds identified from LMA fingerprint.

No.	RT (min)	+TOF-MS (ion) (*m/z*)	Diff (ppm)	−TOF-MS (ion) (*m/z*)	Diff (ppm)	+MS^*n*^ (*m/z*)	−MS^*n*^ (*m/z*)	M.W.	Formula	Identification	Ref.
1	6.448	429.0251 (M + Na)^+^	−6.64	405.0249 (M−H)^−^	0.75	407.1	405.0 ⟶ 190.9 ⟶ 111.1	406	C_21_H_10_O_9_	Citric acid derivative^b^	[8]
2	9.425	330.0609 (M + H)^+^	−0.22	328.0446 (M−H)^−^	5.09	330.1 ⟶ 136.1	328 ⟶ 133.9	329	C_16_H_11_NO_7_	Alkaloid	
3	12.159	346.0545 (M + H)^+^	3.60	344.0407 (M−H)^−^	1.42	346.1 ⟶ 152.1 ⟶ 109.1	343.9 ⟶ 149.9	345	C_16_H_11_NO_8_	Alkaloid	
4	20.457	339.0680 (M + Na)^+^	2.07	315.0706 (M−H)^−^	4.92	339.2⟶185.0	315.0 ⟶ 152.9 ⟶ 109.0	316	C_13_H_16_O_9_	Dihydroxybenzoic acid hexoside^b^	[9]
6	26.642	538.1246 (M + H)^+^	5.05	536.1115 (M−H)^−^	2.55	538.3 ⟶ 334.1 ⟶ 231.1 ⟶ 185.0	536.1 ⟶ 518.1 ⟶ 272.3	537	C_34_H_17_NO_6_	Alkaloid	
8	28.44	339.0689 (M + Na)^+^	−0.78	315.0721 (M−H)^−^	0.18	339.1 ⟶ 254.7 ⟶ 109.2	315.0 ⟶ 152.0 ⟶ 109.0	316	C_13_H_16_O_9_	Dihydroxybenzoic acid hexoside^b^	[9]
11	36.743	539.1375 (M + Na)^+^	−0.72	515.1404 (M−H)^−^	0.44		515.2 ⟶ 178.8 (352.9) ⟶ 135.0	516	C_22_H_28_O_14_	Chlorogenic glycoside^b^	[10]
12	40.471	355.1039 (M + H)^+^	−4.35	353.0871 (M−H)^−^	1.99	355.1 ⟶ 163.1 ⟶ 145.1 ⟶ 117.3	353.0 ⟶ 190.9 ⟶ 172.9	354	C_16_H_18_O_9_	Neochlorogenic acid^ab^	[11]
14	45.428	205.0966 (M + H)^+^	2.71	203.0813 (M−H)^−^	6.38	205.1 ⟶ 188.1	203.0 ⟶ 159.0 ⟶ 129.9	204	C_11_H_12_N_2_O_2_	Tryptophan^ab^	[12]
16	55.956	355.1005 (M + H)^+^	5.25	353.0883 (M−H)^−^	−1.40	355.2 ⟶ 163.1 ⟶ 145.1	353.0 ⟶ 190.9 ⟶ 85.2	354	C_16_H_18_O_9_	Chlorogenic acid^b^	[13]
18	61.352	355.1031 (M + H)^+^	−2.09	353.0887 (M−H)^−^	−2.53	355.1 ⟶ 163.1 ⟶ 145.1	353.0 ⟶ 172.9 (191.0) ⟶ 93.1	354	C_16_H_18_O_9_	4-caffeolyquinic acid^b^	[13]
19	63.725	573.2507 (M + Na)^+^	1.93	595.2632 (M + HCOO)^−^	−4.46	573.4 ⟶ 393.3 ⟶ 362.7	595.3⟶549.3⟶478.9 ⟶ 253.0	550	C_25_H_42_O_13_	Tricalysionoside A^b^	[14]
20	66.36	409.1824 (M + Na)^+^	2.30	431.1907 (M + HCOO)^−^	4.07	409.3 ⟶ 391.2 ⟶ 299.0	431.1⟶178.9 ⟶ 89.2	386	C_19_H_30_O_8_	Roseoside II or isomer^b^	[15]
22	71.239	409.1831 (M + Na)^+^	0.49	431.1950 (M + HCOO)^−^	−7.07	409.3 ⟶ 391.1 ⟶ 334.8	431.1 ⟶ 385.1 ⟶ 153.0 ⟶ 138.0	386	C_19_H_30_O_8_	Roseoside II or isomer^b^	[15]
23	74.107	589.1529 (M + Na)^+^	−0.22	611.1642 (M + HCOO)^−^	−4.31	589.3 ⟶ 427.2 ⟶ 131.2	611.2 ⟶ 565.1⟶ 403.0 ⟶ 240.8	566	C_26_H_30_O_14_	Mulberroside F^b^	[16]
24	76.398	411.1980 (M + Na)^+^	2.42	433.2091 (M + HCOO)^−^	−3.04	411.3 ⟶ 393.3 ⟶ 346.3	433.2 ⟶ 387.1 ⟶ 161.0	388	C_19_H_32_O_8_	Roseoside II or isomer^b^	[15]
25	77.723	633.1408 (M + Na)^+^	2.96	609.1483 (M-H)^−^	−3.59	633.3	609.2 ⟶ 447.0 ⟶ 285.0	610	C_27_H_30_O_16_	Kaempferol-hexoside-hexoside^b^	[11]
26	85.743	465.1052(M + H)^+^	−5.27	463.0866 (M-H)^−^	3.45	465.1	463.1 ⟶ 300.9 ⟶ 272.9	464	C_21_H_20_O_12_	Quercetin hexoside^b^	[17]
28	91.351	611.1641 (M + H)^+^	−5.64	609.1480 (M-H)^−^	−3.10		609.1 ⟶ 299.9 ⟶ 270.9 ⟶ 242.7	610	C_27_H_30_O_16_	Rutin isomer^b^	[11]
30	96.671	611.1615 (M + H)^+^	−1.37	609.1435 (M-H)^−^	4.28	633.3 (611.3) ⟶ 331.2 ⟶ 185.2	609.2 ⟶ 299.9 ⟶ 178.9 ⟶ 150.9	610	C_27_H_30_O_16_	Rutin^ab^	[11]
31	99.07	465.106 (M + H)^+^	−7.00	463.0891 (M-H)^−^	−1.94	465.1 ⟶ 303.1 ⟶ 229.1 ⟶ 201.0	463.1 ⟶ 300.9 ⟶ 150.9 ⟶ 107.1	464	C_21_H_20_O_12_	Isoquercitrin^ab^	[17]
32	104.09	473.1995 (M + Na)^+^	−0.37	449.2031 (M-H)^−^	−0.59	473.3 ⟶ 311.2 ⟶ 249.2	449.2 ⟶ 269.0 ⟶ 207.0 ⟶ 162.9	450	C_20_H_34_O_11_	Unknown	
33	114.084	449.1063 (M + H)^+^	3.43	447.0899 (M-H)^−^	7.50	449.1 ⟶ 287.1 ⟶ 258.1 ⟶ 229.3	447.1 ⟶ 284.0 ⟶ 254.9 ⟶ 188.9	448	C_21_H_20_O_11_	Astragalin^ab^	[11]

^a^The compound was identified by comparison with standard. ^b^The compound was identified by comparison with references.

## Data Availability

The data used to support the findings of this study are available from the corresponding author upon request.
